# Characterization of immune cells in psoriatic adipose tissue

**DOI:** 10.1186/s12967-014-0258-2

**Published:** 2014-09-16

**Authors:** Shawn Rose, Elena Stansky, Pradeep K Dagur, Leigh Samsel, Elizabeth Weiner, Amir Jahanshad, Julia Doveikis, Haley B Naik, Martin P Playford, J Philip McCoy, Nehal N Mehta

**Affiliations:** Section of Inflammation and Cardiometabolic Diseases, National Heart, Lung, and Blood Institute (NHLBI), National Institutes of Health, Bethesda, MD USA; National Institute of Arthritis and Musculoskeletal and Skin Diseases (NIAMS), National Institutes of Health, Bethesda, MD USA; Center for Human Immunology, Autoimmunity, and Inflammation, National Institutes of Health, Bethesda, MD USA; Hematology Branch, NHLBI, National Institutes of Health, Bethesda, MD USA; National Cancer Institute (NCI), National Institutes of Health, Bethesda, MD USA; Cardiovascular and Pulmonary Branch, NHLBI, 10 Center Drive, CRC, Room 5-5140, Bethesda, MD 20892 USA

**Keywords:** Adipose tissue, Immunophenotyping, Imaging flow cytometry, Psoriasis

## Abstract

**Background:**

Adipose tissue normally contains immune cells that regulate adipocyte function and contribute to metabolic disorders including obesity and diabetes mellitus. Psoriasis is associated with increased risk for metabolic disease, which may in part be due to adipose dysfunction, which has not been investigated in psoriasis. There is currently no standardized method for immunophenotyping human adipose tissue. In prior studies, characteristic phenotypic markers of immune cell populations identified in animal models or in other human tissues have been applied in a similar manner to human adipose tissue. Rarely have these populations been verified with confirmatory methodologies or functional studies. Thus, we performed a comprehensive phenotypic and functional analysis of immune cell populations in psoriatic adipose tissue.

**Methods:**

Conventional and imaging flow cytometry were used to define immune cell populations in biopsy specimens of psoriatic adipose tissue (n = 30) including T cells, B cells, NK cells, NKT cells, neutrophils, and macrophages. Relationships between adipose immune cell types and body mass index were determined using Spearman regression analysis, and multivariate linear regression analysis was performed to adjust for cardiometabolic disease risk factors.

**Results:**

These analyses revealed a wide range of cell surface receptors on adipose tissue macrophages, which may serve a dual purpose in immunity and metabolism. Further, both CD16+CD56^Lo^ and CD16-CD56^Hi^ NK cells were found to correlate inversely with body mass index. The relationship between the predominant CD16+CD56^Lo^ NK cell population and body mass index persisted after adjusting for age, sex, diabetes, and tobacco use.

**Conclusions:**

Together, these studies enhance our understanding of adipose immune cell phenotype and function, and demonstrate that examination of adipose tissue may provide greater insight into cardiometabolic pathophysiology in psoriasis.

**Electronic supplementary material:**

The online version of this article (doi:10.1186/s12967-014-0258-2) contains supplementary material, which is available to authorized users.

## Introduction

Adipose tissue plays important roles in energy storage, thermal equilibrium, endocrine function, and immunity [1]. Immune cells within adipose tissue from healthy humans have been implicated in homeostatic functions as well as the initiation and maintenance of metabolic diseases such as obesity and diabetes [2]. Animal models have shown that adipose immune cells contribute to obesity and insulin resistance, including T cells [3–7], B cells [8,9], dendritic cells [10,11], neutrophils [12,13], mast cells [14], and adipose tissue macrophages [ATM] [15–17], while eosinophils [18] and T regulatory cells (Tregs) may protect against insulin resistance [19,20], and NKT cell function is equivocal [21–24]. Adipose specimens from obese humans have demonstrated increased frequencies of T cells [3,25], dendritic cells [10], mast cells [14], neutrophils [26,27], and macrophages [16,28]. In contrast, obese humans have reduced numbers of adipose NKT cells [21] and either an increased [29] or decreased [30] Treg cell mRNA signature compared to lean individuals. CD56+ NK cells have also been demonstrated in human adipose tissue [25,31,32], and the CD16+CD56^Lo^ population has been shown to be reduced in obesity [31].

Immunophenotyping studies of human adipose have generally assumed that characteristic markers of immune cells described in animal models or in other human tissues can be applied similarly to adipose tissue. A major limitation of prior studies has been a paucity of data confirming flow cytometric analysis with alternative methods of cellular identification. Further, the cadre of markers identified on human adipose immune cells remains limited. Here, we have overcome these deficiencies by utilizing cutting-edge conventional and imaging flow cytometry to characterize the immune cell content, phenotype, and function in adipose specimens from patients with the inflammatory skin condition psoriasis, which is associated with an increased risk of cardiometabolic disease (CMD), [33] and adipose tissue dysfunction [34]. We have identified innate and adaptive immune cell populations, and present a panel of ATM markers that may have dual roles in metabolism and immunity. We have also characterized a NK cell population that correlated inversely with body mass index (BMI). Together, these studies are the first to characterize the immune cell repertoire within psoriatic adipose tissue, and may offer insight into the mediators of CMD in psoriasis.

## Methods

### Study population

Subcutaneous gluteal adipose tissue biopsies were performed in psoriasis patients (n = 30) aged 18 to 70 years in a consecutive sample from the Psoriasis Atherosclerosis and Cardiometabolic Disease Initiative (PACI; NCT01778569). A dermatologist confirmed the diagnosis of plaque psoriasis, and performed body surface area (BSA) and Psoriasis Area and Severity Index (PASI) assessments. Psoriatic arthritis was confirmed by a rheumatologist. Exclusion criteria included history of another systemic inflammatory illness, myocardial infarction, stroke, or chronic infectious disease. Study approval was obtained from the National Heart Lung and Blood Institute (NHLBI) institutional review board in accordance with the Declaration of Helsinki. All study participants provided written informed consent.

### Serum factor determination

Fasting levels of total, HDL, and LDL cholesterol, triglycerides, glucose, erythrocyte sedimentation rate (ESR) and high-sensitivity C-reactive protein (hsCRP) were measured in a clinical laboratory.

### Adipose tissue immune cell preparation

Adipose tissue specimens were collected in RPMI medium and minced into fine pieces using scissors after careful removal of infiltrating blood vessels with forceps and scissors. Samples were digested in 5 mg/mL Type IV collagenase (Life Technologies, Grand Island, NY) for 30 minutes at 37°C in an Eppendorf thermomixer (Sigma-Aldrich, St. Louis, MO) at 1300 RPM. Tissue fragments were passed through a 40 μM nylon filter (BD Falcon, Beford, MA), washed twice with 1X PBS, and the floating adipocyte fraction was removed by vacuum aspiration. Blood contamination was determined by the visible presence of large amounts of erythrocytes in the cell pellet after digestion and centrifugation. Further, blood contaminated samples demonstrated abundant granulocyte populations by flow cytometry that are not typically present in adipose tissue. Three adipose samples were excluded from the analyses due to blood contamination.

### Conventional flow cytometry

Adipose immune cells were stained with various cocktails (up to 15-parameter) of surface antibodies in staining buffer [1X PBS without calcium or magnesium containing 5% heat-inactivated fetal bovine serum (Atlas, Fort Collins, CO), 0.25 mM EDTA (Sigma-Aldrich), and 0.09% sodium azide (Sigma-Aldrich)] for 30 minutes at 4°C. A 1:500 solution of LIVE/DEAD Aqua (Life Technologies) was added during the last 10 minutes of staining. For samples requiring intracellular staining, cells were fixed and permeabilized using BD Cytofix/Cytoperm kits (BD Biosciences, San Jose, CA) according to the manufacturer’s instructions, followed by intracellular staining for 30 minutes at 4°C. T cell IFN-γ and TNF-α production was elicited by incubation with 10 ng/mL PMA (BD Biosciences) and 1 μg/mL ionomycin (BD Biosciences) in the presence of 10 μg/mL brefeldin A (BD Biosciences) for 3 hours prior to intracellular cytokine staining. FoxP3, granzyme B, IL-1α, and IL-8 were assessed in unstimulated cells without using a protein transport inhibitor. Foxp3 staining was conducted similarly to other intracellular antigens, except that Foxp3 staining kits (eBioscience, San Diego, CA) were used. Positive staining for each antibody-fluorochrome combination was determined using fluorescence minus one (FMO) controls. Additional file 1: Table S3 lists the antibodies utilized for conventional and imaging flow cytometry. Samples were acquired on a BD Biosciences LSR II flow cytometer equipped with 405 nm, 488 nm, 532 nm, and 633 nm excitation lasers using DIVA software (BD Biosciences). Compensation was performed with single color controls prepared using BD Biosciences CompBeads. Compensation matrices were calculated automatically followed by manual adjustment and sample analysis using FlowJo software (Tree Star, Ashland, OR).

### Phagocytosis assays

Peripheral blood mononuclear cells (PBMC) were prepared over a Ficoll gradient using standard methods. CD14+ monocytes were positively selected from PBMC or adipose biopsy specimens utilizing CD14 MicroBeads (Miltenyi Biotech, San Diego, CA), followed by passage over MACS LS columns (Miltenyi Biotech) according to the manufacturer’s instructions. Enrichment of CD14+ ATM was ~6-fold compared to ~20-fold enrichment of CD14+ monocytes from PBMC. Phagocytosis assays were performed on CD14+ monocytes, CD14+ enriched adipose specimens, and whole adipose specimens using the pHrodo (Life Technologies) system. Cells were incubated (≥5 x 10^5^ per well) in 96-well plates containing complete DMEM medium (Life Technologies) for 1.5 hours at 37°C. Culture supernatants were removed by aspiration, and opsonized pHrodo *S. aureus* bioparticles (Life Technologies) were added to the cells for 1.5 hours at either 37°C or 4°C (negative control). Cells were washed in staining buffer and stained for surface antigens prior to flow cytometric analysis.

### Imaging flow cytometry

Surface staining was performed as described above. Cells were washed with 1X PBS buffer containing 0.5 mM EDTA and 0.2% BSA at pH 7.2, suspended at a concentration of 1–5 × 10^6^/mL, and then incubated in 0.1 mM Hoechst (Life Technologies) at 37°C for 30 minutes. Positive staining for each antibody-fluorochrome combination was determined using FMO controls. Samples were acquired on an Amnis ImageStream X Mark II instrument equipped with 405 nM, 488 nM, 561 nM, and 640 nM lasers utilizing INSPIRE software (Amnis, Seattle, WA). Automatic compensation was performed with single color controls (BD Comp Beads), followed by manual adjustment and analysis using IDEAS 6.0 software (Amnis).

### Statistical analysis

Spearman correlations were performed between adipose NK Cell frequencies and BMI, and multivariate linear regression was used to adjust for CMD risk factors (age, sex, diabetes, and tobacco use) and for treatment with oral corticosteroids, disease-modifying anti-rheumatic drugs (DMARDs), and/or biologic agents. No significant effects of treatment were identified. Thus, we report results from multivariate linear regression modeling after adjustment for CMD risk factors. Kruskall-Wallis testing with post-hoc Dunn’s multiple comparisons testing was performed to compare MFI values for surface markers among ATM populations. Adipose cell populations and cytokine expression were compared between psoriasis and control patients using Mann–Whitney U tests. Significance was considered at p < 0.05. Statistical tests were performed using Graphpad Prism (LaJolla, CA) and STATA (College Station, TX) software.

## Results

### Patient demographics and clinical evaluation

Patient characteristics (n = 30) and laboratory measurements are presented in Table 1. Our study population had a median age of 54 years [interquartile range (IQR) 41–61], was 54% male, had a median BMI of 29 (IQR 25.9-32.3), had moderate psoriasis (mean BSA 9.2 ± 16, mean PASI score 7.8 ± 9.3), and 38% had psoriatic arthritis (Table 1). Medication usage and CMD were also assessed. Topical steroid use was common (37%) and 3 patients received phototherapy (Table 1). Biologic therapy (39%) was more common than DMARD (9%) treatment (Table 1). Hypertension (32%), dyslipidemia (68%), diabetes (11%), and tobacco use (9% active, 28% former) were prevalent in our study population (Table 1), as was treatment for hypertension (19%), dyslipidemia (37%), and diabetes (6%).

**Table 1:** **Patient characteristics**

**(n = 30)**	**Median (IQR)**
Age (years)	54 (41–61)
Male, count (%)	35 (54)
Psoriasis Disease Duration (years)	20 (9–32)
Body Surface Area Score [Mean (SD)]	9.2 (16)
PASI Score [Mean (SD)]	7.8 (9.3)
Psoriatic Arthritis, count (%)	25 (38)
DMARD Therapy, count (%)	6 (9)
Biologic Therapy, count (%)	25 (39)
NSAID Therapy, count (%)	15 (23)
Phototherapy, count (%)	3 (5)
Topical Steroid Therapy, count (%)	24 (37)
Systemic Steroid Therapy, count (%)	1 (2)
Diabetes Mellitus, count (%)	7 (11)
Hypertension, count (%)	21 (32)
Dyslipidemia, count (%)	44 (68)
Current Tobacco Use, count (%)	6 (9)
Former Tobacco Use, count (%)	18 (28)
Diabetes Mellitus Therapy, count (%)	4 (6)
Anti-Hypertensive Therapy, count (%)	12 (19)
Dyslipidemia therapy, count (%)	24 (37)
Body Mass Index (kg/m2)	29 (25.9-32.3)
Systolic Blood Pressure (mm Hg)	125 (116–135)
Diastolic Blood Pressure (mm Hg)	72 (65–78)
Fasting Blood Glucose (mg/dL)	94 (89–104)
Total Cholesterol (mg/dL)	184 (158–203)
Triglycerides (mg/dL)	108 (84–137)
High-Density Lipoprotein Cholesterol (mg/dL)	52 (42–63)
Low-Density Lipoprotein Cholesterol (mg/dL)	96 (80–125)
Erythrocyte Sedimentation Rate (mm/hr)	8 (5–13)
High-Sensitivity C-Reactive Protein (g/dL)	1.7 (0.7-4.2)

IQR = Interquartile Range, PASI = Psoriasis Area and Severity Index, DMARD = Disease-Modifying Anti-Rheumatic Drug, NSAID = Non-Steroidal Anti-Inflammatory Drug. Data are reported as median (IQR) unless indicated otherwise.

DMARD therapy denotes methotrexate use, except for 1 patient who was taking both methotrexate and hydroxychloroquine. Biologic therapy indicates active TNF antagonist or anti-IL-12/23 receptor use except for one patient who was treated with abatacept for psoriatic arthritis.

### Adipose immune cell characterization

**Figure 1:**
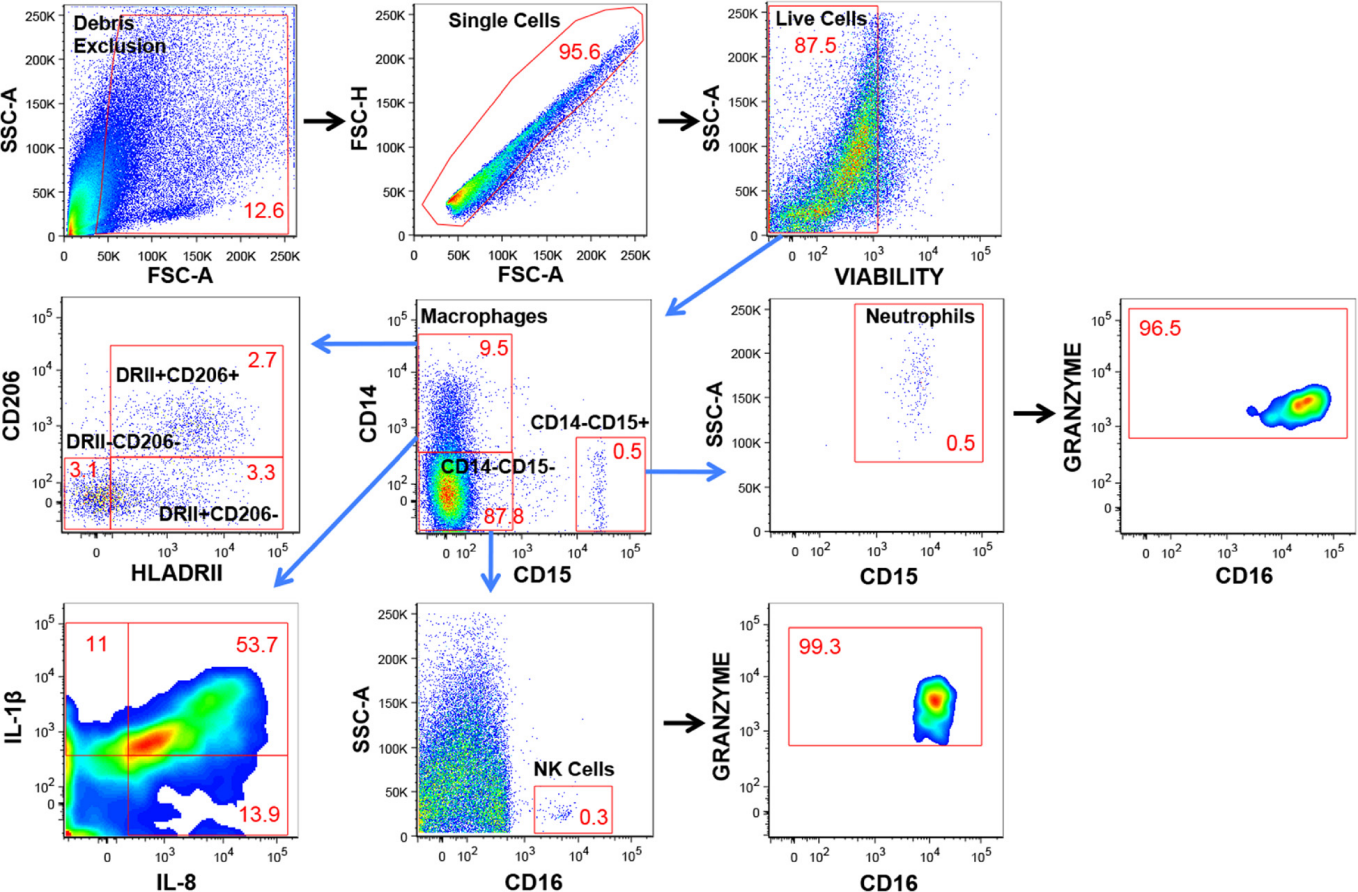
**Flow cytometric characterization of macrophages, NK cells, and neutrophils in a representative sample of psoriatic adipose tissue.** After exclusion of debris, doublets, and non-viable cells, cells were identified as follows: neutrophils = CD14-CD15+CD16+SSC^Hi^granzyme B+, NK cells = CD14-CD15-CD16+/−CD56+/−SSC^lo^granzyme B+, adipose tissue macrophages (ATM) = CD14+CD15-CD16-, which were further gated into 3 subpopulations based on HLADRII and CD206 expression. IL-1β and IL-8 intracellular staining is presented for the total ATM population. All cell populations are presented as percentages of viable cells except for IL-1β and/or IL-8 expressing ATM, and Granzyme B expressing neutrophils, which are reported as percentages of the parent population. Positive gating for each fluorochrome parameter was established using individual fluorescence minus one (FMO) controls.

**Figure 2:**
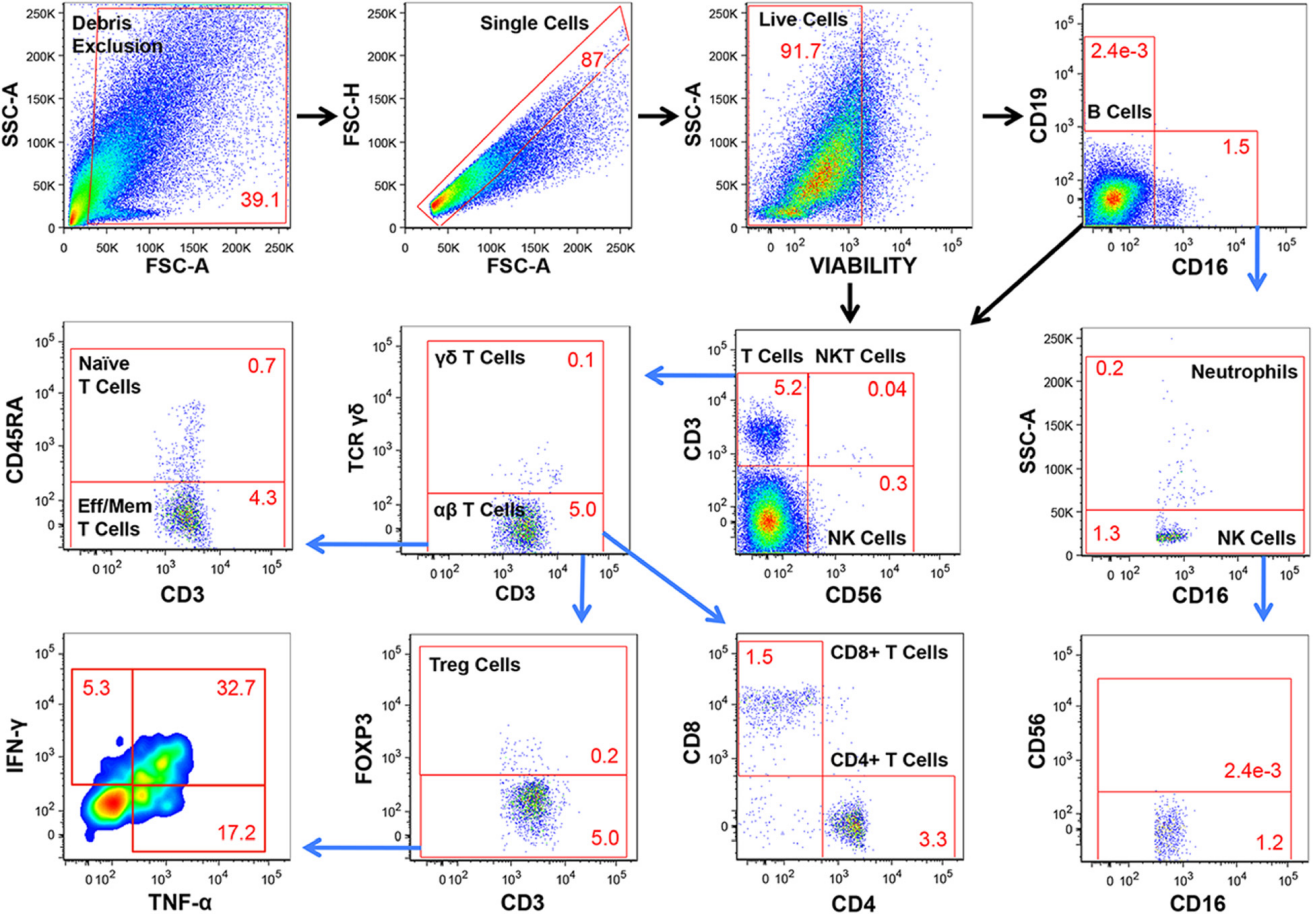
**Flow cytometric characterization of B cells, T cells, NK cells, NKT cells, and neutrophils in a representative sample of psoriatic adipose tissue.** After exclusion of debris, doublets, and non-viable cells, CD16-CD19+ B cells, CD3+CD56- T cells, CD3-CD16-CD56^Hi^ NK cells, CD3+CD56+ NKT cells, and total CD16+ cells were identified. CD16+ cells were divided into neutrophils (CD16+SSC^Hi^) and NK cells (CD16+CD56^Lo^SSC^Lo^) based on SSC properties. CD3+ T cells were further gated into naïve and effector and/or memory (eff/mem) αβ T cell subsets, CD4+CD8- T cells, CD4-CD8+ T cells, γδ T cells, and T regulatory (FoxP3+) cells. All cell populations are presented as percentages of viable cells except for granzyme B, IFN-γ, and TNF-α expressing cells, which are reported as percentages of the parent population. Positive gating for each fluorochrome parameter was established using FMO controls.

Multi-parameter flow cytometry was applied to psoriatic adipose tissue, and gating strategies for cell identification (Figures 1, 2) and immune cell frequencies (Additional file 2: Table S1) are presented. Together, ATM (CD3-CD14+CD15-CD16-CD19-CD56-, Figure 1) were the most numerous immune cell type, comprising ~10% of viable cells. ATM made the prototypical cytokines IL-1β and IL-8 (Figure 1). Three populations of CD14+ ATM were identified based on HLADRII and CD206 expression (Figure 1). The HLADRII+CD206- subset was more abundant than the HLADRII-CD206- and HLADRII+CD206+ subsets. Frequencies of IL-1β and IL-8 expressing cells were not statistically different among the ATM subsets, except that the percentage of HLADRII+CD206-IL-8+ cells was significantly greater (p < 0.05) than HLADRII-CD206-IL-8+ cells (Additional file 3: Table S2). T cells (CD3+CD14-CD15-CD16-CD19-CD56-, Figure 2) collectively made up ~4.5% of viable cells, and were primarily αβ T cells with a predominance of CD4+ T cells. αβ T cells were largely either effector or memory cells (CD45RA-, Figure 2) and made the T cell cytokines IFN-γ and TNF-α (Figure 2). CD8+ T cells, FoxP3+ Tregs (predominately CD4+, Additional file 4: Figure S1), γδ T cells, and NKT cells were also identifiable (Figure 2). Confirmation of NKT cell (CD3+CD14-CD15-CD16-CD19-CD56+SSC^lo^, Figure 2) phenotype was attempted using alpha-gal-cer loaded CD1d tetramers, but the scarcity of this population precluded definitive characterization. NK cells (CD3-CD14-CD15-CD16+/−CD19-CD56^Hi/Lo^SSC^lo^, Figures 1, 2) comprised ~1.5% of viable cells, were largely CD16+CD56^Lo^ versus CD16-CD56^Hi^, and expressed the characteristic protease granzyme B (Figure 2). B cells (CD3-CD14-CD15-CD16-CD19+CD56-PD-1+, Figure 2, Additional file 5: Figure S2) made up <1% of viable cells. Neutrophils (CD3-CD14-CD15+CD16 + CD19-CD56-SSC^Hi^, Figures 1, 2) were also infrequent and expressed the characteristic marker granzyme B (Figure 2).

### Verification of adipose tissue macrophage phenotype and function

**Figure 3:**
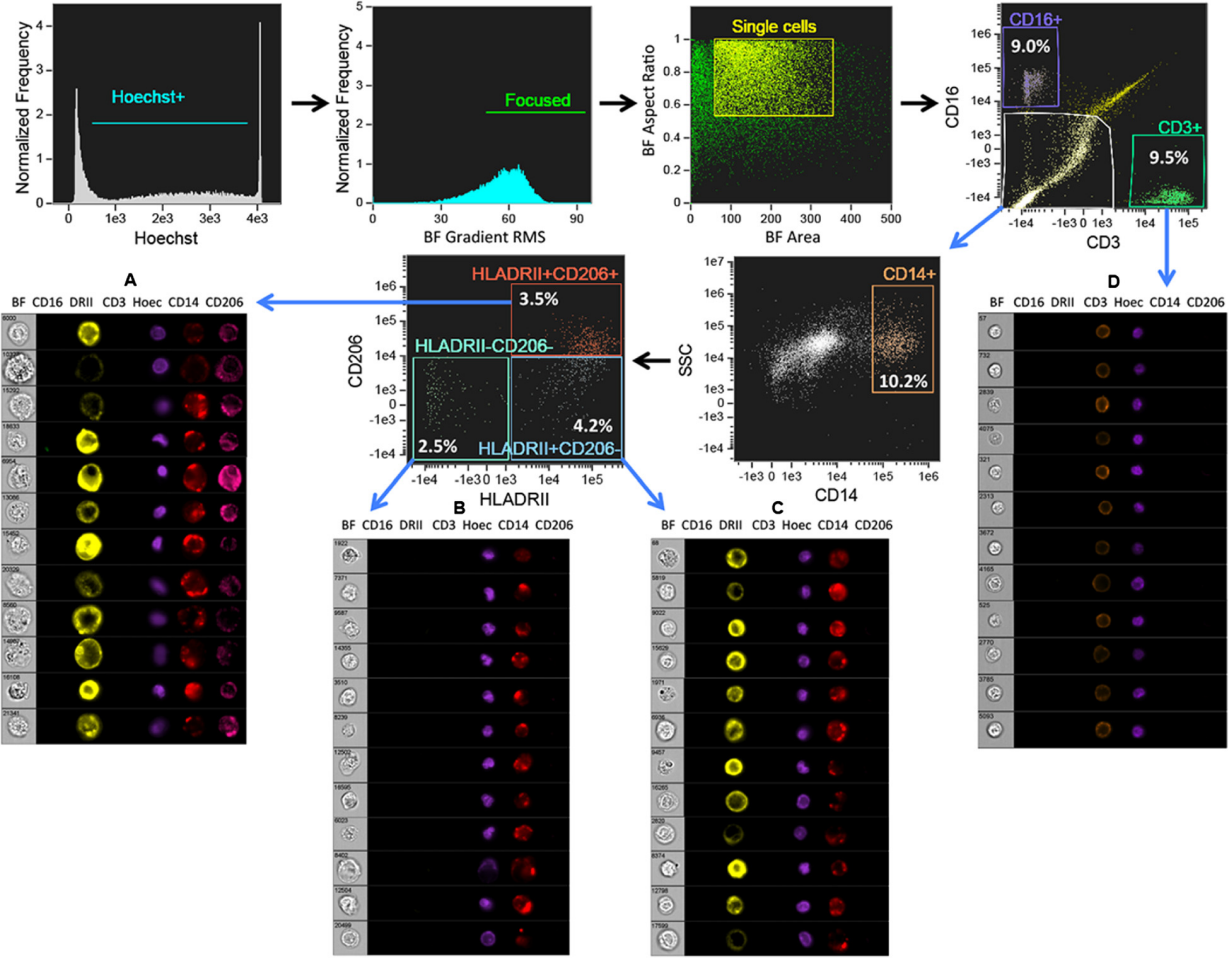
**Confirmation of immune cell populations in psoriatic adipose tissue using imaging flow cytometry.** Morphologic and staining characteristics of various cell populations delineated by conventional flow cytometry (Figures 1 and 2) were confirmed in psoriatic adipose tissue using imaging flow cytometry. Cells were gated for positive nuclear staining that did not saturate the camera. Out-of-focus cells were excluded using the gradient RMS function of the brightfield microscopy field. Doublets and debris were eliminated by plotting cell brightfield area against aspect ratio. To determine nuclear morphology population characteristics, manually selected mononuclear or polymorphonuclear cells were identified and hand tagged populations were created. The Feature Finder wizard in IDEAS software was then used to distinguish cells with similar morphologic characteristics in the source population using the circularity and bright detail intensity features on the Hoechst imagery. Three populations of cells were identified: CD3+CD16- T cells **(panel D)**, CD3-CD16+ cells, and CD3-CD16- cells. CD3-CD16- cells were then gated on CD14+ cells (macrophages), followed by sub-gating based on CD206 and HLADRII (DRII) staining **(panels A-C)**. Positive gating for each fluorochrome parameter was established using FMO controls. Percentages of cells in each gate are presented as percentages of nucleated, focused, single cells.

**Figure 4:**
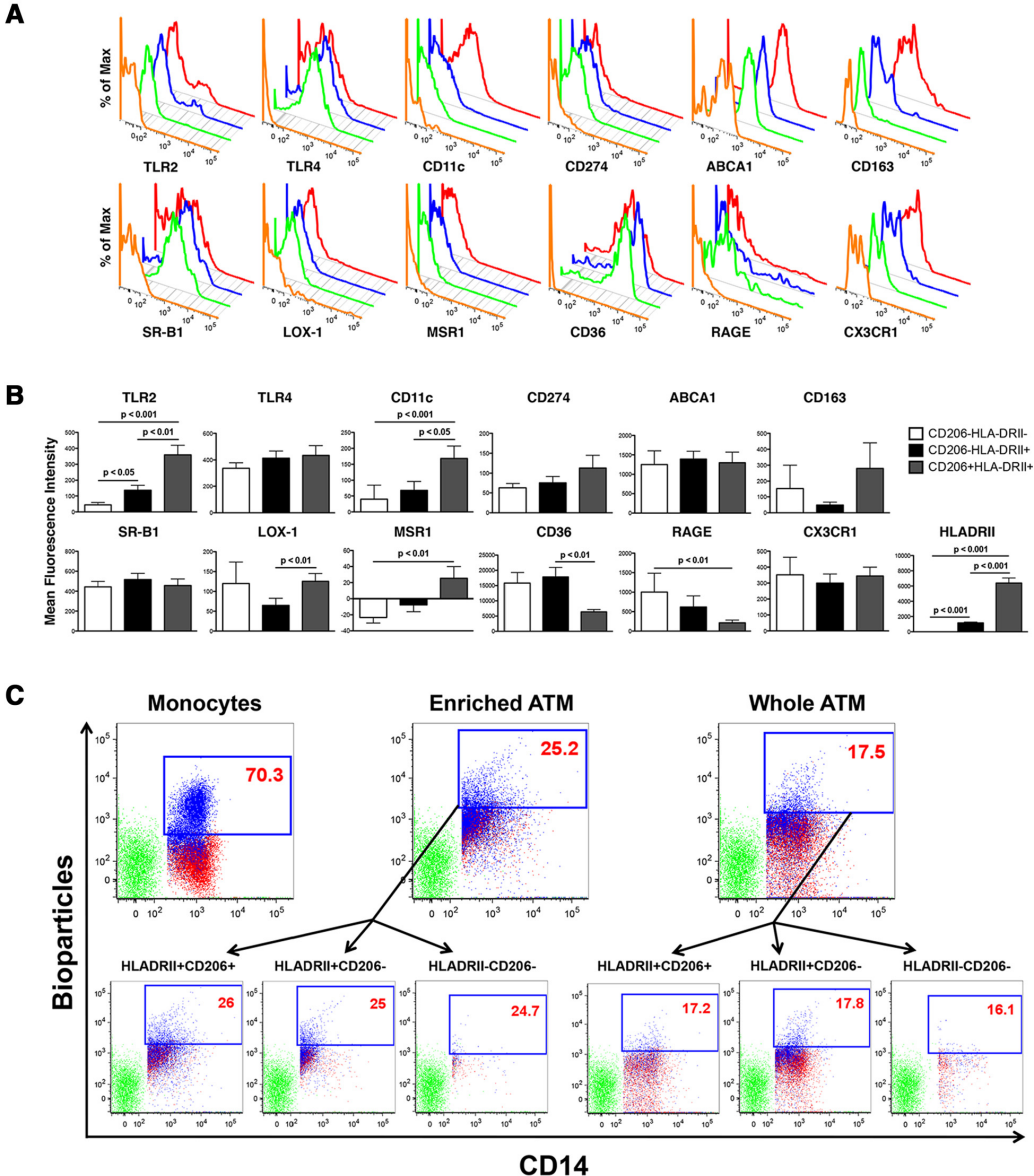
**Flow cytometric analysis of surface receptor expression and phagocytic function in adipose tissue macrophages. (A)** ATM populations were examined for expression of TLR2, TLR4, CD11c, CD274, ABCA1, CD163, SR-B1, LOX-1, MSR1, CD36, RAGE, and CX3CR1. CD14+HLADRII-CD206- (green histograms), CD14+HLADRII+CD206- (blue histograms), and CD14+HLADRII+CD206+ (red histograms) cells are presented compared to FMO control staining for each marker (orange histograms). **(B)** Mean fluorescence intensity values for each surface marker depicted in **(A)** were compared in CD14+HLADRII-CD206-, CD14+HLADRII+CD206-, and CD14+HLADRII+CD206+ ATM. Kruskall-Wallis testing with post-hoc Dunn’s multiple comparison testing was performed to determine whether surface macrophage marker expression was statistically different among the different subsets. p values < 0.05 were considered to be statistically significant. **(C)** The pHrodo system was utilized to demonstrate phagocytosis of opsonized bioparticles by enriched CD14+ monocytes (Monocytes, left panel, positive control), positively selected CD14+ ATM (Enriched ATM, center panel), and whole adipose tissue, gated on CD14+ ATM (Whole ATM, right panel) by flow cytometry. Green dots represent bioparticles incubated with antibody staining cocktail and no cells (acellular negative control). Red dots represent monocytes or ATM incubated with bioparticles at 4°C (cellular negative control). Blue dots represent monocytes or ATM incubated with bioparticles at 37°C. Enriched ATM and Whole ATM were further gated into 3 subpopulations based on HLADRII and CD206 expression. Phagocytosis of opsonized bioparticles is depicted for each subpopulation of Enriched ATM and Whole ATM and is reported as percentages of viable cells containing bioparticles.

Conventional flow cytometry demonstrated various immune cell subsets in psoriatic adipose tissue using classic phenotypic markers. The ability of these markers to correctly identify adipose immune cells was confirmed by imaging flow cytometry. This technology combines features of brightfield microscopy, flow cytometry, and immunofluorescence microscopy in a single instrument, thus allowing for high-resolution characterization of cell populations in tissues [35]. Imaging flow cytometry verified ATM by positive CD14 staining, abundant cytoplasm, and round to U-shaped nuclei consistent with macrophage morphology (Figure 3, panels A-C). Three subsets of ATM were present based on HLADRII and CD206 expression (Figure 3), and ATM rarely expressed CD16 (mean 1.47 ± 1.77%, Additional file 6: Figure S3). Conventional flow cytometry was then performed to uncover ATM markers that may link metabolism and immunity in psoriasis (Figure 4). ATM expressed a wide array of surface receptors with roles in microbial pattern recognition (TLR2, TLR4), immune suppression (CD274), cell adhesion (CD11c), cholesterol trafficking (ABCA1), chemokine binding (CX3CR1), and macromolecule clearance (CD163, SR-B1, LOX-1, MSR1, RAGE, and CD36) (Figure 4A). Mean fluorescence intensity (MFI) values for these receptors were strikingly different among the macrophage subpopulations (Figure 4B). TLR2, CD11c, MSR1, and HLADRII expression were highest in HLADRII+CD206+, intermediate in HLADRII+CD206-, and lowest in HLADRII-CD206- ATM (Figure 4B). LOX-1 expression was lowest in the HLADRII+CD206- population, CD36 expression was lowest in the HLADRII+CD206+ subset, and RAGE expression was highest in HLADRII-CD206-, intermediate in HLADRII+CD206-, and lowest in HLADRII+CD206+ cells (Figure 4B). ATM phagocytic function was also assessed by flow cytometry using the pHrodo system, which utilizes bioparticles that fluoresce only upon acidification within phagocyte endosomes [36]. Phagocytosis was equivalent among the 3 ATM subpopulations (Figure 4C).

### Verification of other adipose immune cell populations

**Figure 5:**
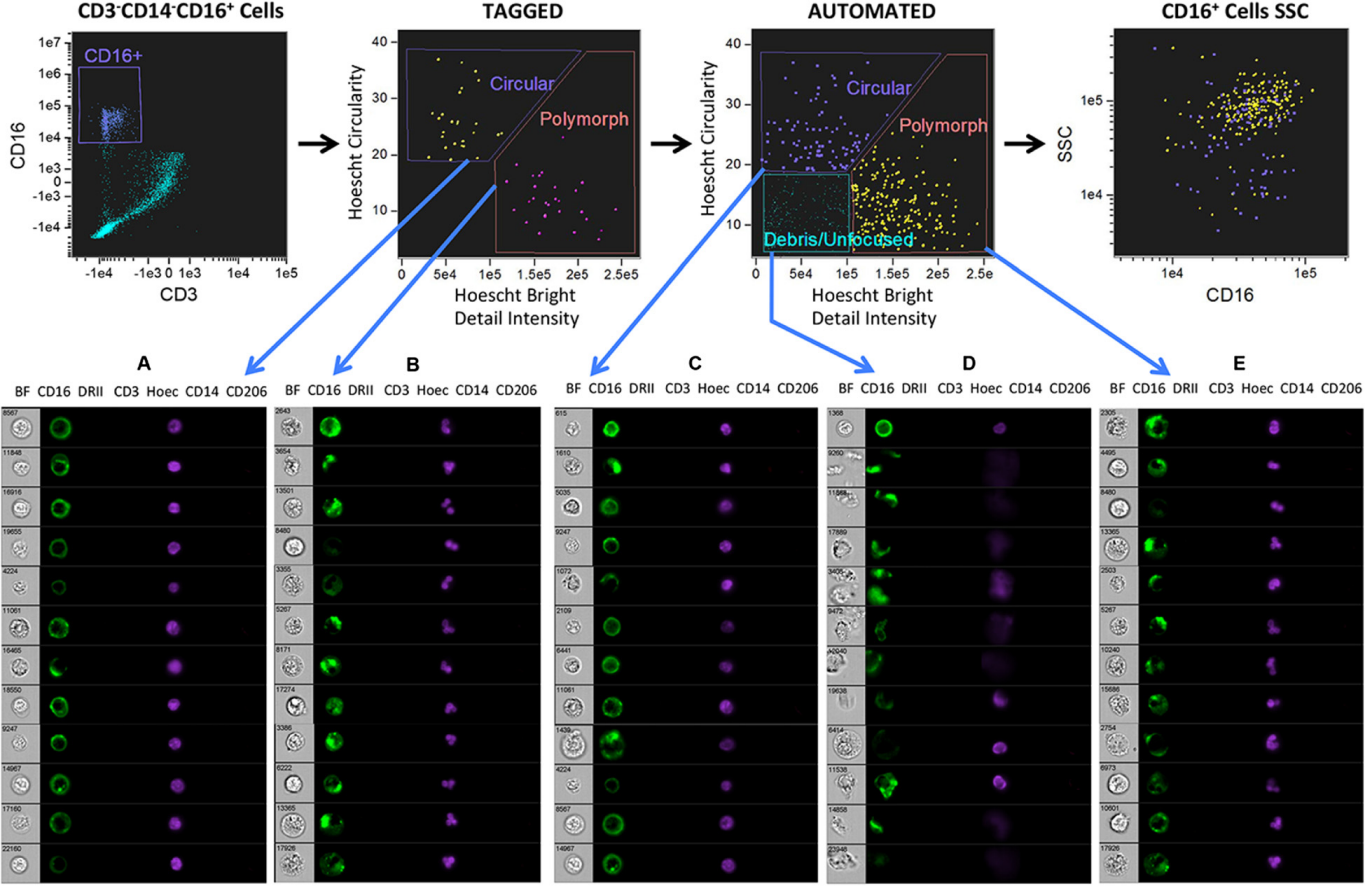
**Nuclear morphologic analysis of CD16+ cell populations in adipose tissue confirms flow cytometric phenotypic analysis.** Imaging flow cytometry of adipose tissue immune cells was performed as in Figure 3. After exclusion of non-nucleated cells and Hoechst saturating the camera, poorly focused cells, and debris/doublets, CD3-CD14- cells were gated on CD16+ cells. CD16+ cells (~30 cells for each group) were manually selected (Tagged) based on their nuclear morphology to distinguish mononuclear (Circular, panel **A**) from polymorphonuclear (Polymorph, panel **B**) cells. The Feature Finder wizard in IDEAS software was used to identify similar cells in the total CD16+ source population (Automated, panels **C**, **E**) and to exclude debris and unfocused cells (Automated, panel **D**). Circularity and Bright Detail Intensity of the Hoechst imagery were the 2 characteristics that best distinguished the manually selected Polymorph and Circular cells. A plot of these 2 parameters for the Tagged and Automated CD16+ populations is depicted. BF = brightfield microscopy, Hoec = Hoechst nuclear staining. CD16 was plotted against SSC to compare Circular and Polymorph populations. Positive gating for each fluorochrome parameter was established using FMO controls.

Imaging flow cytometry confirmed other immune cell populations in psoriatic adipose tissue. T cells were verified by positive CD3 staining, round nuclear morphology, and scant cytoplasm (Figure 3, panel D). To determine cell types that express CD16 in adipose tissue, CD3-CD14-CD16+ cells were manually categorized (hand tagged) as having mononuclear (circular, Figure 5, panel A) or polymorphic (polymorph, Figure 5, panel B) nuclei. The Feature Finder wizard in IDEAS software was then used to distinguish cells from the source population with similar morphologic characteristics. This strategy allowed segregation of cellular debris/unfocused cells (Figure 5, panel D), mononuclear cells (Figure 5, panel C), and polymorphonuclear cells (Figure 5, panel E) within the CD16+ population. CD16+ polymorphonuclear cells had multi-lobed (2–3 lobes) nuclei and dense granularity, characteristic of neutrophils. In contrast, CD16+ mononuclear cells had circular nuclei and variable granularity, confirming their identity as NK cells. Further, CD16+ NK cells did not express the macrophage markers HLADRII or CD206 (Additional file 6: Figure S3), nor did they express IL-1β, ACBA1, TLR2, TLR4, CD274, LOX-1, MSR1, CD36, RAGE, CD163, or SR-B1 (Additional file 7: Figure S4).

### Psoriatic adipose NK cells are inversely related to obesity

**Figure 6:**
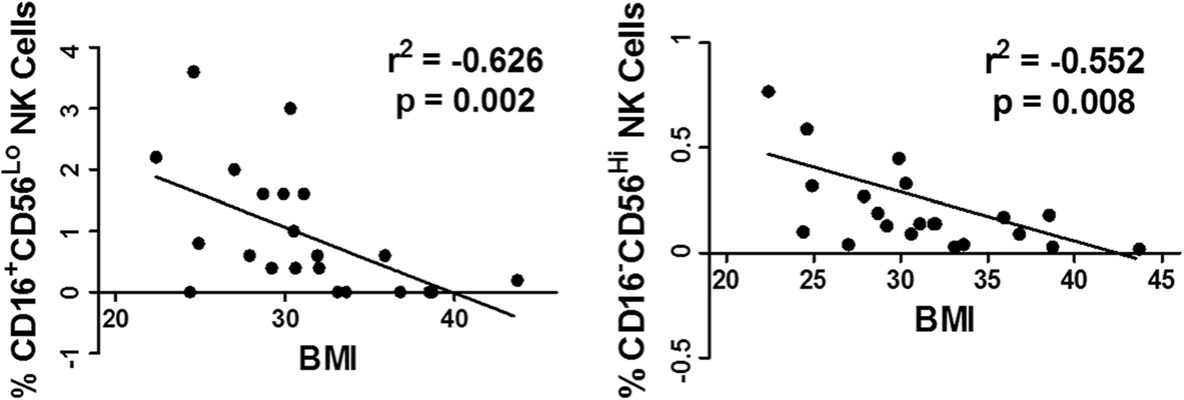
**NK Cell frequencies in psoriatic adipose tissue are inversely correlated with BMI.** Multi-parameter flow cytometry was performed as in Figure 1. Relationships between frequencies of CD16+CD56^Lo^ (left panel) or CD16-CD56^Hi^ (right panel) NK cells (x-axis) and BMI (y-axis) were determined using Spearman regression analysis. Regression plots and corresponding r^2^ and p values are depicted.

To better understand how adipose immune cells relate to obesity in psoriasis, we performed Spearman correlation analyses between individual immune cell subset frequencies and BMI. Both CD16-CD56^Hi^ (r −0.552, p = 0.008) and CD16+CD56^Lo^ NK cells (r −0.626, p = 0.002) were inversely correlated with BMI in unadjusted analyses (Figure 6). The inverse relationship between BMI (outcome variable) and adipose CD16+CD56^Lo^ NK cells remained significant after adjusting for the CMD risk factors age, sex, diabetes, and tobacco use (β -46.5, p = 0.04), and with additional adjustment for treatment with oral corticosteroids, DMARDs, and/or biologics (β -54.04, p = 0.026). In contrast, BMI and CD16-CD56^Hi^ NK cell frequencies were no longer associated after adjustment for CMD risk factors (β -1.89, p = 0.172). BMI was also unrelated to the other adipose immune cell types.

### Comparison of adipose immune cells in psoriasis and controls

To determine whether adipose immune cell composition is affected by psoriasis, we performed a nested case–control study (n = 6 non-diabetic control and psoriasis patients) matched for age, sex, BMI, smoking, and dyslipidemia. We observed that CD16-CD56^Hi^ NK cells, but not other immune cells, were statistically greater (0.42% live cells in psoriasis versus 0.06% live cells in controls, p = 0.014) in psoriatic compared to control adipose. While the frequencies of total ATM were similar in psoriasis and controls (10.2% live cells in psoriasis versus 12.9% live cells in controls versus, p = 1.0), IL-1β producing (46.1% of psoriatic ATM versus 29.3% of control ATM, p = 0.076) and IL-8 producing (66.1% of psoriatic ATM versus 51.0% of control ATM, p = 0.175) ATM were more abundant in psoriatic compared to control adipose tissue. However, only the HLADRII+CD206+ ATM subset demonstrated a statistically significant difference in IL-1β (40.3% in psoriasis versus 19.6% in controls, p = 0.047), but not IL-8 (62.5% in psoriasis versus 45.7% in controls, p = 0.117), production between psoriatic and control adipose tissue.

## Discussion

We utilized cutting-edge imaging technologies to demonstrate that psoriatic adipose tissue contains immune cells that may influence CMD in psoriasis. Using this approach, we have: (1) identified previously unappreciated immune cell populations and cell surface receptors (2) utilized a combination of phenotypic markers and functional studies to validate those populations (3) described an inverse relationship between psoriatic adipose CD16+CD56^Lo^ NK cells and obesity. We believe these studies take a critical first step toward understanding CMD in psoriasis, and may lead to novel therapies targeting these disorders.

Here, we distinguished 3 populations of ATM expressing varying levels of multi-functional receptors (CD36, LOX-1, MSR1, RAGE, and TLR2) that may perform dual roles in immunity and metabolism. For example, the scavenger receptors MSR1 [37], CD36 [38], and LOX-1 [39] have been shown to bind modified LDL particles, which play a prominent role in CMD and coronary artery disease [40–43]. Furthermore, mRNA levels of MSR1, CD36, and LOX-1 in whole adipose tissue specimens have been associated with obesity and insulin resistance in humans [44]. These pleiotropic scavenger receptors also demonstrate important functions in innate immune defense [45]. Similarly, RAGE [46,47] and TLR2 [48,49] have essential roles in immunity and CMD. Thus, our data support a growing body of literature indicating that macrophages may directly modulate adipocyte function [50]. Our findings also suggest that ATM populations may represent unique functional subsets that respond to different stimuli, and extend well beyond the oversimplified M1/M2 macrophage paradigm [51–53]. This notion could be addressed by exploring global gene expression patterns from purified ATM populations both in the basal state and after activation with various ligands. Importantly, we also demonstrated that psoriatic ATM may be predisposed toward pro-inflammatory cytokine expression, which could contribute to adipose dysfunction in this disorder.

Several other populations of immune cells were identified using conventional and imaging flow cytometry. We are the first to quantify frequencies of FoxP3+ Tregs [previously reported by FoxP3 mRNA expression [29,30]] and γδ T cells in human adipose tissue. It is also noteworthy that CD16+ cells were primarily neutrophils and NK cells. Bourlier et al. previously found that up to 25% of ATM express CD16 [51]. However, control staining for CD16 was not presented in their manuscript. In our experience, staining with anti-CD16 antibodies in adipose tissue can give high background signal and thus requires careful antibody titration, Fc Receptor saturation, and appropriate control stains. Both conventional and imaging flow cytometric analyses confirmed that CD16 expression on ATM is rare. Low frequencies of B cells [25], NKT cells [21], and neutrophils [26,27] were also apparent in psoriatic adipose tissue, confirming prior studies in humans. Adipose CD3+ T cells were predominantly αβ T cells and were largely either effector or memory in phenotype, as previously reported [25]. Consistent with previous reports [3,25], CD4+ T cells were well represented followed in descending order of frequency by CD8+ T cells, Tregs, and γδ T cells. The nature of the cognate antigens driving T cell priming locally and/or prior to migration into adipose tissue remains to be determined.

Adipose NK cells may contribute to obesity in humans. Two populations of NK cells have previously been identified, and are designated as CD16-CD56^Hi^ [25,31,32] and CD16+CD56^Lo^ [31] cells. We demonstrated that the predominant CD16+CD56^Lo^ NK cell population correlated inversely with BMI in both unadjusted analyses and after adjustment for CMD risk factors and psoriasis treatment. These findings corroborate those of O’Rourke et al., who demonstrated that the CD16+CD56^Lo^ subset was reduced in obese compared to lean adipose tissue [31]. In contrast, these investigators found increased percentages of CD16-CD56^Hi^ cells in adipose tissue from obese compared to lean patients [31], while Duffaut and colleagues reported no differences in CD56+ NK cells (CD16 expression was not analyzed) irrespective of BMI [25]. Despite these inconsistencies, there is an inverse relationship between adiposity and the major population of adipose NK cells (CD16+CD56^Lo^). Future studies in animal models should directly test whether NK cell deficiency impacts adipose tissue composition and function. Two indirect lines of evidence have suggested this to be the case. First, studies of the NK cell growth factor IL-15 have shown that IL-15 overexpression increased adipose NK cell infiltration and decreased adipose tissue mass in mice [54], although the latter outcome may have been due to a direct effect of IL-15 on adipocytes [55]. Second, leptin receptor mutant animals demonstrated lower circulating and tissue NK cell numbers and impaired NK cell function as well as prominent obesity and insulin resistance [56]. Together, these studies suggest that NK cells may protect against obesity in mouse and man.

We acknowledge that this study had certain limitations such as modest sample size and single-center study design, which could affect the generalizability of the results. However, we provide unique data in 30 psoriatic patients under standardized study conditions (NCT01778569). Another consideration is that therapies for psoriasis and/or CMD could impact adipose tissue cell populations. Our study used subcutaneous adipose tissue from psoriasis patients. While visceral adipose tissue is generally considered to be more inflamed, recent studies have revealed that subcutaneous and visceral adipose display a similar pro-inflammatory phenotype, suggesting that experiments using the readily accessible subcutaneous depot will enhance our understanding of adipose biology [57,58]. Despite these limitations, our data are methodologically sound and may be informative for understanding CMD in psoriasis.

## Conclusions

1. Advanced immunophenotyping will enhance our understanding of adipose immune cell composition and function in health and disease.
2. Immune cell characterization of psoriatic adipose tissue may provide greater insight into CMD pathophysiology in psoriasis.

## Additional files

Additional file 1: Table S3. Antibodies Used for Flow Cytometry Experiments. Additional file 2: Table S1. Summary of immune cell populations in psoriatic adipose tissue. Additional file 3: Table S2. IL-1β and IL-8 Expression by Adipose Tissue Macrophages. Additional file 4: Figure S1. The majority of Tregs in psoriatic adipose tissue are CD4+. Multi-parameter flow cytometry was performed as in Figure 2. After sequentially gating out debris, doublets, and non-viable cells, Treg cells were identified as CD3+CD16-CD19-CD56-TCRγδ-FoxP3+ cells. CD4+ and CD8+ Tregs from a representative sample are depicted with cell frequencies presented as percentages of the parent population. Positive gating for each fluorochrome parameter was established using FMO controls. Additional file 5: Figure S2. PD-1 is highly expressed on adipose tissue B cells. Multi-parameter flow cytometry was performed as in Figure 2. After sequentially gating out debris, doublets, and non-viable cells, B cells were identified as CD3-CD16-CD19+CD56- cells with high surface expression of the B cell marker PD-1. A representative sample is depicted, with cell frequencies presented as percentages of the parent population. Positive gating for each fluorochrome parameter was established using FMO controls. Additional file 6: Figure S3. Specificity of adipose tissue macrophage markers. Imaging flow cytometric analysis of psoriatic adipose tissue was performed to determine the morphologic and staining characteristics of CD16+ cells delineated by flow cytometry (Figures 1 and 2). After exclusion of non-nucleated cells and Hoechst saturating the camera, poorly focused cells, and debris/doublets, CD3-CD14- cells were sub-gated based on HLADRII (DRII) and CD206 staining. CD3-CD14+ cells were also examined for CD16 expression. Cell frequencies are presented as percentages of nucleated, focused, single cells. Positive gating for each fluorochrome parameter was established using FMO controls. Additional file 7: Figure S4. NK Cell phenotyping in psoriatic adipose tissue. Multi-parameter flow cytometry was performed as in Figures 1 and 2. After sequentially gating out debris, doublets, and non-viable cells, the vast majority (91-99%) of NK cells were identified as CD3-CD14-CD16+CD19-CD56^Lo^HLADRII- cells. NK cells were analyzed for ABCA1, IL-1β, TLR2, TLR4, CD274, LOX-1, MSR1, CD36, RAGE, CD163, and SR-B1 expression. NK cells from a representative sample are depicted with cell frequencies presented as percentages of the parent population. CD16+CD56^Lo^ NK cells (red histograms) are presented compared to FMO controls (blue histograms) for each fluorochrome parameter.

## Abbreviations

ATM: Adipose tissue macrophages
BMI: Body mass index
BSA: Body surface area
CMD: Cardiometabolic disease
DMARD: Disease-modifying anti-rheumatic drug
ESR: Erythrocyte sedimentation rate
hsCRP: High-sensitivity C-reactive protein
HDL: High-density lipoprotein
LDL: Low-density lipoprotein
MFI: Mean fluorescence intensity
PASI: Psoriasis area and severity index
