# Supplementary material for: Characterization of immune cells in psoriatic adipose tissue
Source: J Transl Med. 2014 Sep 16;12:258. doi: 10.1186/s12967-014-0258-2 (PMC4197293; doi:10.1186/s12967-014-0258-2)
Supplement: Additional file 1: Table S3. — Antibodies Used for Flow Cytometry Experiments. [file 12967_2014_258_MOESM1_ESM.doc]

**Additional file 1: Table S3. Antibodies Used for Flow Cytometry Experiments**

| **Antigen** | **Clone** | **Fluorochrome** | **Company** | **Experiment** |
| --- | --- | --- | --- | --- |
| ABCA1 | NB400-105F | FITC | Novus Biologicals | FC |
| CD1d Tetramers | D001-2 | APC* | Proimmune | FC |
| CD3 | UCHT1 | Brilliant Violet 421 | Biolegend | FC |
| CD3 | UCHT1 | PE-CF594 | BD Biosciences | IS |
| CD4 | OKT4 | Brilliant Violet 655 | Biolegend | FC |
| CD8 | RPA-T8 | Brilliant Violet 605 | Biolegend | FC |
| CD8 | RPA-T8 | PE | BD Biosciences | FC |
| CD11c | 3.9 | AlexaFluor 700 | eBioscience | FC |
| CD14 | 61D3 | eFluor 605NC | eBioscience | FC |
| CD14 | 61D3 | APC | eBioscience | IS |
| CD15 | HI98 | PE-CF594 | BD Biosciences | FC |
| CD16 | 3G8 | Brilliant Violet 711 | Biolegend | FC |
| CD16 | 3G8 | AlexaFluor 700 | BD Biosciences | FC |
| CD16 | CB16 | FITC | eBioscience | IS |
| CD19 | HIB19 | Brilliant Violet 785 | Biolegend | FC |
| CD36 | CB38 | V450 | BD Biosciences | FC |
| CD45RA | HI100 | PE-CF594 | BD Biosciences | FC |
| CD56 | HCD56 | PerCP-Cy5.5 | Biolegend | FC |
| CD163 | GHI/61 | PE | eBioscience | FC |
| CD206 | 15-2 | APC-Cy7 | Biolegend | FC, IS |
| CD274 (PD-L1) | MIH1 | PerCP-eFluor 710 | eBioscience | FC |
| CD279 (PD-1) | EH12.2H7 | Brilliant Violet 711 | Biolegend | FC |
| CX3CR1 | 2A9-1 | APC | Biolegend | FC |
| FOXP3 | 236A/E7 | PE | eBioscience | FC |
| Granzyme B | GB11 | AlexaFluor 700 | BD Biosciences | FC |
| HLA-DRII | L243 | Brilliant Violet 785 | Biolegend | FC |
| HLA-DRII | L243 | PE | Biolegend | FC |
| IL-1β | JK1B-1 | AlexaFluor 647 | Biolegend | FC |
| IL-8 | G265-8 | PE | BD Biosciences | FC |
| LOX-1 | 331212 | APC | R&D Systems | FC |
| MSR1 | 351615 | AlexaFluor 700 | R&D Systems | FC |
| RAGE | ab54741 | eFluor 655NC** | Abcam | FC |
| SRB-1 | NB400-104R | Dylight 550 | Novus Biologicals | FC |
| TCRγδ | B1 | FITC | Biolegend | FC |
| TLR2 | T2.5 | AlexaFluor 488 | Biolegend | FC |
| TLR4 | HTA-125 | PE-Cy7 | eBioscience | FC |

FC = Flow Cytometry, IS = ImageStream, *denotes pre-conjugated with alpha-gal-cer

**denotes conjugated in our laboratory
