# Supplementary material for: Characterization of immune cells in psoriatic adipose tissue
Source: J Transl Med. 2014 Sep 16;12:258. doi: 10.1186/s12967-014-0258-2 (PMC4197293; doi:10.1186/s12967-014-0258-2)
Supplement: Additional file 2: Table S1. — Summary of immune cell populations in psoriatic adipose tissue. [file 12967_2014_258_MOESM2_ESM.doc]

| **Additional file 2: Table S1. Summary of immune cell populations in psoriatic adipose tissue.** | | | |
| --- | --- | --- | --- |
| **Cell Type** | **Mean Frequency** | **SD** | **N** |
| HLADRII+CD206- ATM | 4.79 | 5.16 | 30 |
| HLADRII-CD206- ATM | 3.75 | 3.08 | 30 |
| HLADRII+CD206+ ATM | 1.51 | 1.54 | 30 |
| αβ T Cells | 4.22 | 2.60 | 22 |
| CD4+ T Cells | 2.33 | 1.57 | 22 |
| Memory αβ T Cells | 3.60 | 2.21 | 22 |
| Naïve αβ T Cells | 0.63 | 0.50 | 22 |
| CD8+ T Cells | 1.39 | 1.08 | 22 |
| FoxP3+ Tregs | 0.41 | 0.22 | 22 |
| γδ T Cells | 0.27 | 0.23 | 22 |
| NKT Cells | 0.05 | 0.06 | 22 |
| CD16+CD56^Lo^NK Cells | 1.24 | 0.89 | 22 |
| CD16-CD56^Hi^ NK Cells | 0.26 | 0.34 | 22 |
| B Cells | 0.97 | 1.47 | 22 |
| Neutrophils | 0.47 | 0.77 | 30 |
| Immune cell populations were identified by flow cytometry as demonstrated in Figures 1a and 1b. Frequencies of cells are reported as mean ± standard deviation (SD) percentage of viable cells for each cell population. N denotes number of patients evaluated. ATM denotes adipose tissue macrophages. | | | |
|  | | | |
|  | | | |
|  | | | |
