# Supplementary material for: Characterization of immune cells in psoriatic adipose tissue
Source: J Transl Med. 2014 Sep 16;12:258. doi: 10.1186/s12967-014-0258-2 (PMC4197293; doi:10.1186/s12967-014-0258-2)
Supplement: Additional file 3: Table S2. — IL-1β and IL-8 Expression by Adipose Tissue Macrophages. [file 12967_2014_258_MOESM3_ESM.doc]

**Additional file 3: Table S2. IL-1β and IL-8 Expression by Adipose Tissue Macrophages**

| **Cell Phenotype** | **% IL-1β Expressors** | **% IL-8 Expressors** |
| --- | --- | --- |
| HLADRII-CD206- ATM | 38.1 ± 22.4 | 55.3 ± 20.2* |
| HLADRII+CD206- ATM | 44.0 ± 17.9 | 70.2 ± 18.0 |
| HLADRII+CD206+ ATM | 35.0 ± 16.0 | 57.7 ± 22.7 |

Intracellular cytokine staining was performed to determine the mean ± standard deviation frequencies of IL-1β and IL-8 expressing CD14+ adipose tissue macrophage (ATM) subpopulations as defined in Figure 1a. Frequencies of cytokine expressing cells were compared among groups (n=20 for each group) using Kruskall-Wallis testing with post-hoc Dunn’s Multiple Comparison testing. *denotes statistically significant difference (p<0.05) between the HLADRII-CD206- and HLADRII+CD206- population.
