# Supplementary figures and images for: Characterization of immune cells in psoriatic adipose tissue
Source: J Transl Med. 2014 Sep 16;12:258. doi: 10.1186/s12967-014-0258-2 (PMC4197293; doi:10.1186/s12967-014-0258-2)

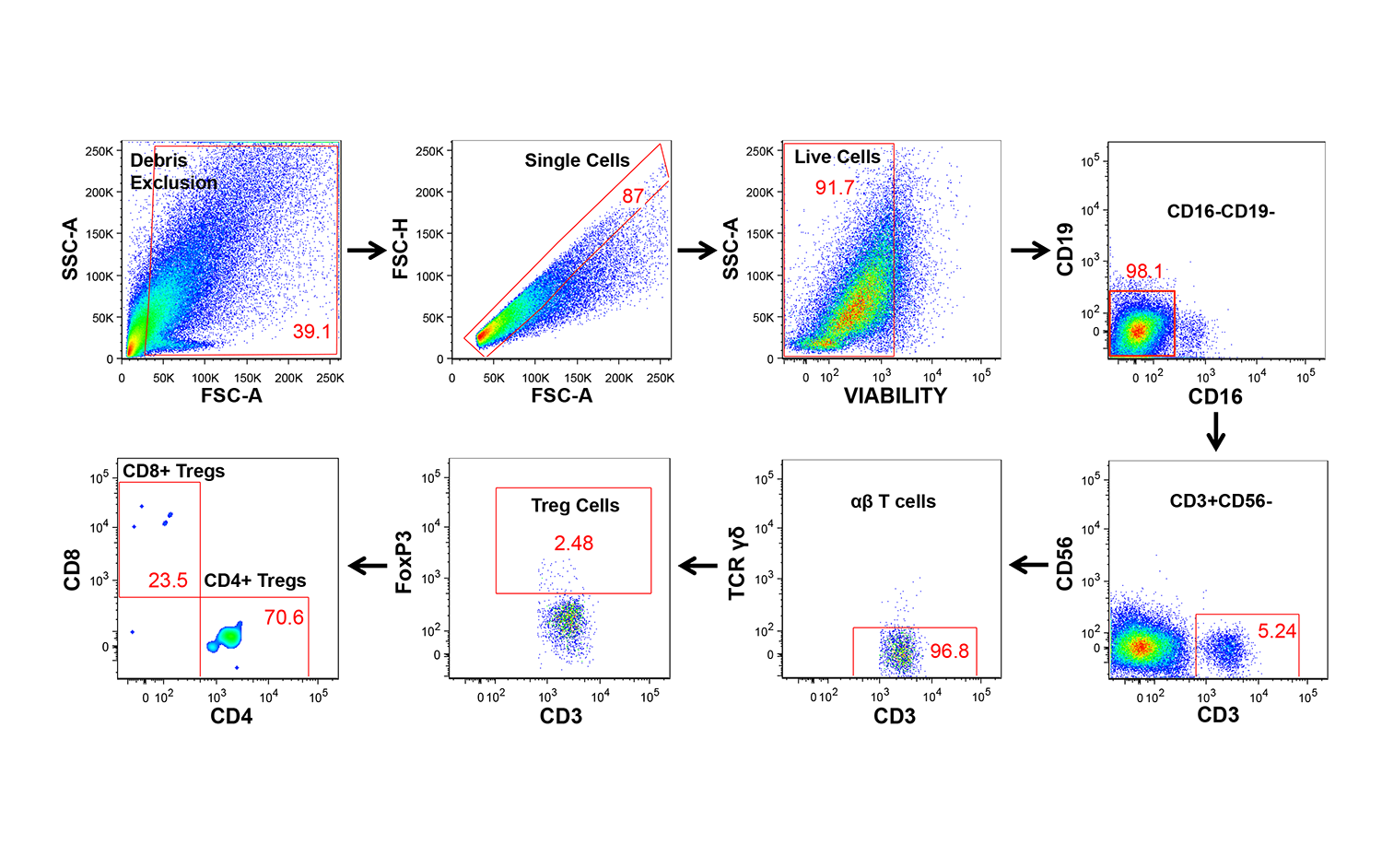

Supplement: Additional file 4: Figure S1. — The majority of Tregs in psoriatic adipose tissue are CD4+. Multi-parameter flow cytometry was performed as in Figure 2. After sequentially gating out debris, doublets, and non-viable cells, Treg cells were identified as CD3+CD16-CD19-CD56-TCRγδ-FoxP3+ cells. CD4+ and CD8+ Tregs from a representative sample are depicted with cell frequencies presented as percentages of the parent population. Positive gating for each fluorochrome parameter was established using FMO controls. [file 12967_2014_258_MOESM4_ESM.tiff]

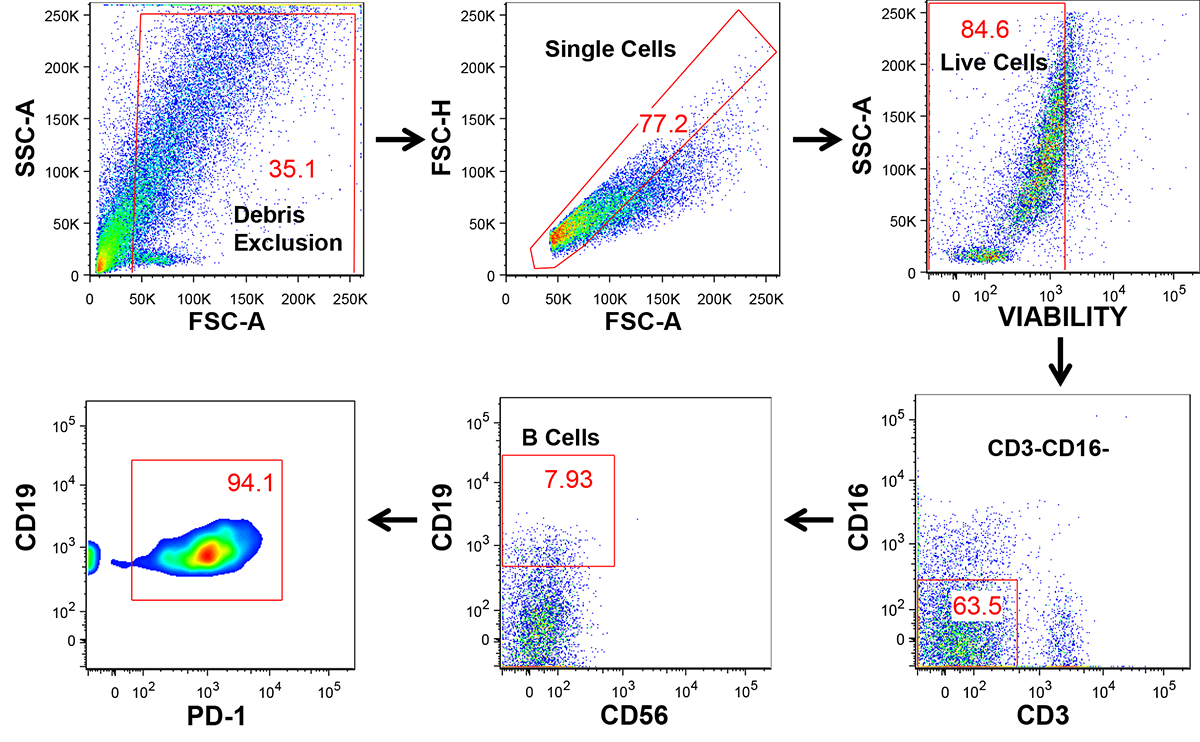

Supplement: Additional file 5: Figure S2. — PD-1 is highly expressed on adipose tissue B cells. Multi-parameter flow cytometry was performed as in Figure 2. After sequentially gating out debris, doublets, and non-viable cells, B cells were identified as CD3-CD16-CD19+CD56- cells with high surface expression of the B cell marker PD-1. A representative sample is depicted, with cell frequencies presented as percentages of the parent population. Positive gating for each fluorochrome parameter was established using FMO controls. [file 12967_2014_258_MOESM5_ESM.tiff]

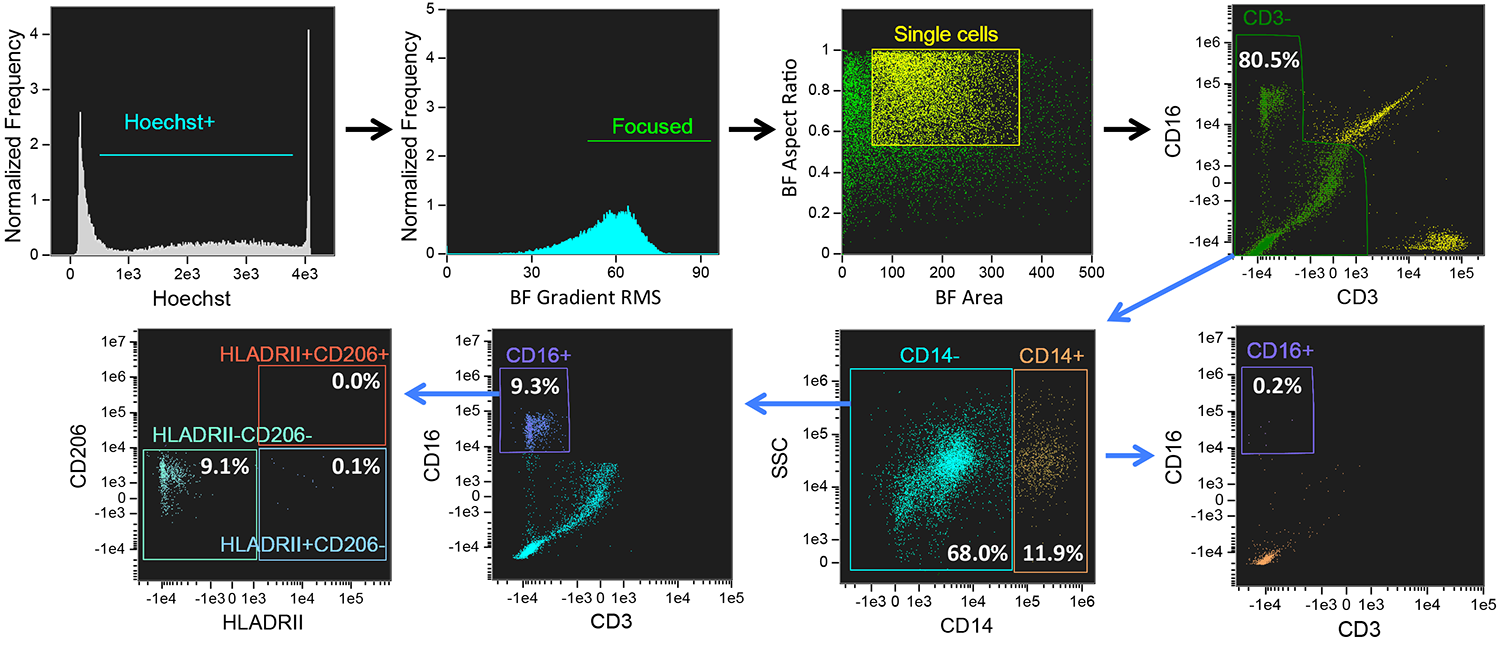

Supplement: Additional file 6: Figure S3. — Specificity of adipose tissue macrophage markers. Imaging flow cytometric analysis of psoriatic adipose tissue was performed to determine the morphologic and staining characteristics of CD16+ cells delineated by flow cytometry (Figures 1 and 2). After exclusion of non-nucleated cells and Hoechst saturating the camera, poorly focused cells, and debris/doublets, CD3-CD14- cells were sub-gated based on HLADRII (DRII) and CD206 staining. CD3-CD14+ cells were also examined for CD16 expression. Cell frequencies are presented as percentages of nucleated, focused, single cells. Positive gating for each fluorochrome parameter was established using FMO controls. [file 12967_2014_258_MOESM6_ESM.tiff]

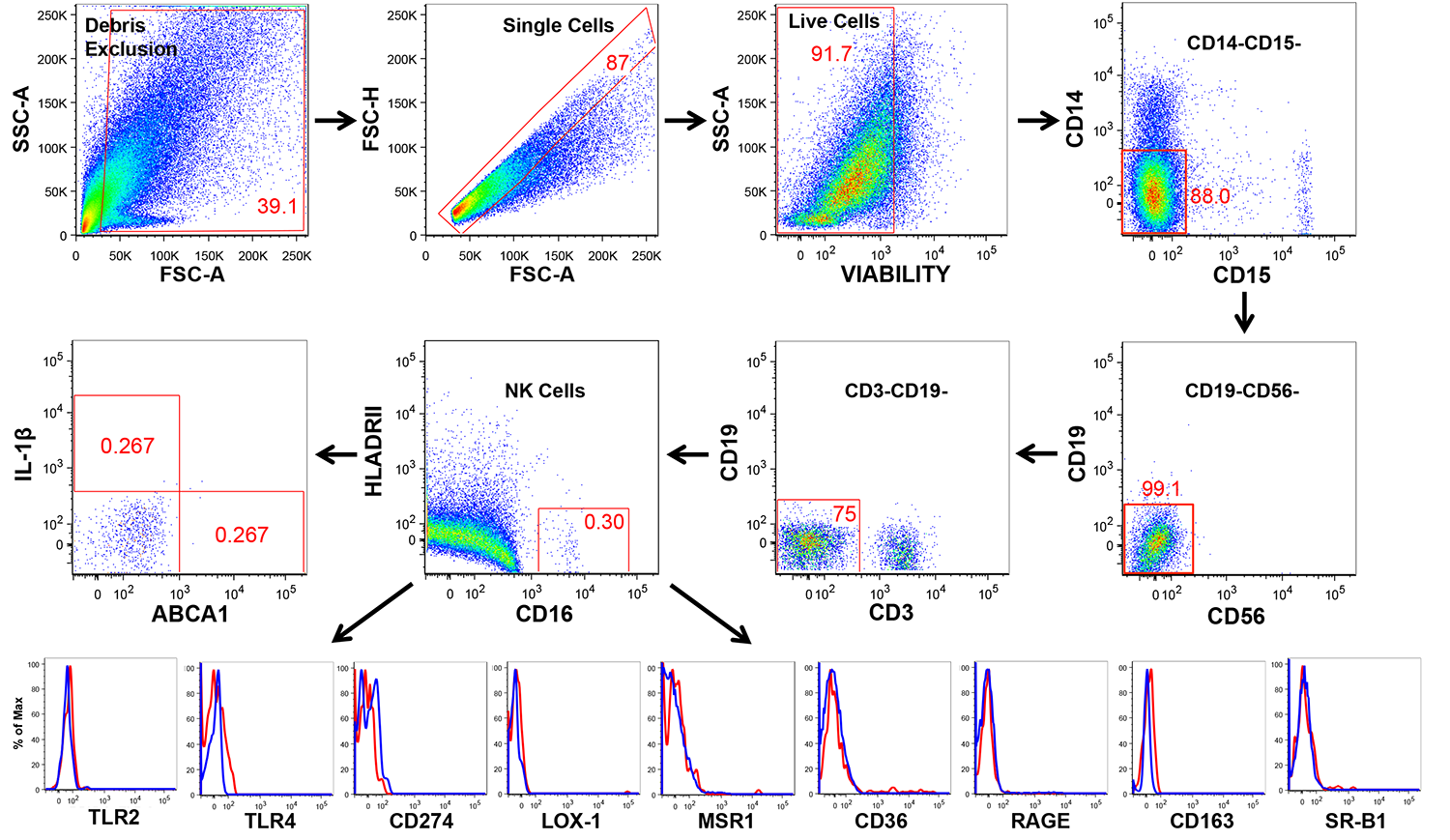

Supplement: Additional file 7: Figure S4. — NK Cell phenotyping in psoriatic adipose tissue. Multi-parameter flow cytometry was performed as in Figures 1 and 2. After sequentially gating out debris, doublets, and non-viable cells, the vast majority (91-99%) of NK cells were identified as CD3-CD14-CD16+CD19-CD56LoHLADRII- cells. NK cells were analyzed for ABCA1, IL-1β, TLR2, TLR4, CD274, LOX-1, MSR1, CD36, RAGE, CD163, and SR-B1 expression. NK cells from a representative sample are depicted with cell frequencies presented as percentages of the parent population. CD16+CD56Lo NK cells (red histograms) are presented compared to FMO controls (blue histograms) for each fluorochrome parameter. [file 12967_2014_258_MOESM7_ESM.tiff]
